# Effect of dipeptidyl peptidase-4 inhibitors inhibitor on cognitive dysfunction in diabetes: A protocol for systematic review and meta analysis

**DOI:** 10.1097/MD.0000000000020707

**Published:** 2020-07-31

**Authors:** Shiyu Liu, Xian Wang, Huan Deng, Yuguo Xia, Xiaomei Yang, Qiu Chen

**Affiliations:** a School of Clinical Medicine; b Department of Endocrinology, Hospital of Chengdu University of Traditional Chinese Medicine, Chengdu, China.

**Keywords:** cognitive dysfunction, dipeptidyl peptidase IV, incretin glucagon-like peptide-4, type 2 diabetes

## Abstract

**Introduction:**

There is a risk of cognitive impairment in diabetic patients. Some studies have shown that dipeptidyl peptidase-4 inhibitors (DPP-4) inhibitors can play a protective role in controlling blood glucose and blocking the DPP-4 to attach antibody glucagon-like peptide -1 degradation and prolong antibody glucagon-like peptide -1, promoting the growth of neurites and the formation of synapses. The purpose of this study is to explore the effect of the DPP-4 inhibitor on cognitive impairment in diabetic patients by meta-analysis.

**Methods:**

The system review plan will strictly follow the Systematic Review and Meta-Analysis Protocols entry for reporting. PubMed, EMBASE, Cochrane Library, Clinicaltrials(clinicaltrials.gov), Web of Science, China National Knowledge Infrastructure, China Science and Technology Journal Database and Wanfang Databases will be systematically searched, and all randomized controlled trials comparing DPP-4 inhibitors with placebo or other hypoglycemic drugs to study cognitive impairment in type 2 diabetic patients will be included. The inclusion, evaluation and data extraction of the literature will be conducted by 2 persons independently, and the dispute will be resolved by a third person. All the meta-analysis of the included literature and the research progress of the existing research are analyzed as the main results.

**Ethics and dissemination:**

It is to evaluate and analyze the completed research, so there is no ethical problem. The research results will be published in a peer-reviewed journal.

**Registration:**

The protocol of this systematic review and meta-analysis was registered on International Platform of Registered Systematic Review and Meta-analysis Protocols (https://inplasy.com/) (number. 202040185).

## Abstract 2

Supplemental Digital Content is available in the text

## 1 Introduction

Type 2 diabetes (T2DM) is a cognitive risk factor and is associated with mild cognitive impairment (MCI), Alzheimer's disease, dementia and other cognitive disorders.^[1]^ Although the etiology of cognitive impairment in people with T2DM is uncertain,^[2]^ but people with diabetes have a greater rate of decline in cognitive function and a greater risk of cognitive decline.^[3]^ which reduce quality of life and increase social burden.^[4]^ Due to the large population of patients with diabetes, and effective treatment for cognitive impairment has not yet been found,^[5,6]^ it is necessary to search for effective drugs.

Increased dipeptidyl peptidase-4 inhibitors (DPP4) activities are independently associated with MCI in elderly patients with T2DM. The possible reason is that it has an impact on some risk factors related to MCI, so it may be the therapeutic target of MCI.^[7]^ As an oral hypoglycemic agent for T2DM, DPP-4, an incretin glucagon-like peptide-1 (GLP-1) degrading enzyme, in diabetic use, the DPP-4 inhibitor can block the DDP-4 to attenuate GLP-1 degradation and prolong GLP-1 its action and sensitize insulin activity for the purpose of lowering blood glucose.^[8]^ GLP-1 was shown to act as a neurotrophic factor and protect against neurodegeneration, possibly by promoting long-term potentiation, enhancing neurite outgrowth and contributing to synapse formation, in a manner that resembles nerve growth factor.^[9]^

A study demonstrated that augmentation of GLP-1 by DPP-4 inhibitors enhances glucose-induced insulin secretion and decreases glucagon secretion over a daily period, as well as reduces hemoglobin A1c (HbA1c) and glycemic fluctuations over a daily period.^[10]^ In an animal experiment, Sitagliptin, a dipeptidyl peptidase-4 inhibitor, exhibit dual benefits by improving metabolic control and reducing the decline in cognitive function.^[11]^ A retrospective longitudinal clinical trial found the addition of vildagliptin to treatment, improved the copying subdomain of cognitive function and metabolic control of the older patients with T2DM within 6 months.^[12]^ Another retrospective also shows that vildagliptin in addition to metformin showing a protecting role on cognitive functioning compared to the metformin only group in elderly diabetic patients with MCI.^[13]^ In a Real-World Population-Based Cohort Study, DPP-4i used decreases the risk of dementia compared to sulfonylureas (SU) used in elderly patients with T2DM.^[14]^

These studies seem to show the positive effects of DPP-4 inhibitors, but still controversial,^[15]^ and there is no systematic evaluation of the use of DPP-4 inhibitors in cognitive dysfunction in diabetes, therefore, it is valuable in exploring the evidence regarding the use to better understand its potential clinical usefulness.

## 2 Objectives

This systematic review aims to identify and critically evaluate randomized controlled trials (RCT) of DPP-4 inhibitors in cognitive dysfunction in T2DM. A comprehensive understanding of the current level of evidence of the literature will useful for providing new ideas about the cognitive problems of diabetic patients targetedly and inform future research.

## 3 Methods and analysis

Before writing the protocol, we searched relevant databases and found no published or registered system evaluation and meta-analysis. This systematic review will be conducted in accordance with the Prefer Reporting Items for Systematic Reviews and Meta-Analyses protocols guidelines.^[16]^ The study was registered on International Platform of Registered Systematic Review and Meta-analysis Protocols (https://inplasy.com/) in April 25, 2020 with registration number 202040185. We will systematically conduct literature retrieval, and meta-analysis will be carried out after obtaining sufficient data. If there is not enough quantity or quality of randomized controlled trials, only systematic evaluation will be carried out.

### 3.1 Inclusion and exclusion criteria

Objects that meet all the following conditions: RCT subjects include patients with T2DM; the experimental group received DPP4 inhibitors (listing drugs, including sitagliptin, saxagliptin, vildagliptin, ritagliptin, and aglitin.etc); the control group received placebo or other hypoglycemic agents; the patients’ cognitive function was evaluated before and after the experiment.

We will exclude studies with any of the following characteristics: The study reported that the control group's plan was unclear; the intervention group's medication, dosage, frequency, and duration were unclear; the data on cognitive function scores could not be extracted separately; documents not written in Chinese or English will be excluded.

### 3.2 Search strategy

The PICOS (population, intervention/exposure, comparison/control, Outcome, study design) framework was used to develop the search strategy for this review. Medical subheadings words and key words will be used to search PubMed, EMBASE, Cochrane Library, clinical trails.gov, Web of Science, China National Knowledge Infrastructure, China Science, the technical journal database and Wanfang database for documents. Restricted to search “cognitive impairment”, “dementia”, “type 2 diabetes” in the title and abstract, and restricted to “Mental State and Dementia Test” in the full text. An example of Cochran's search strategy is shown in Supplementary material Table 1.

### 3.3 Study selection

**Figure 1:**
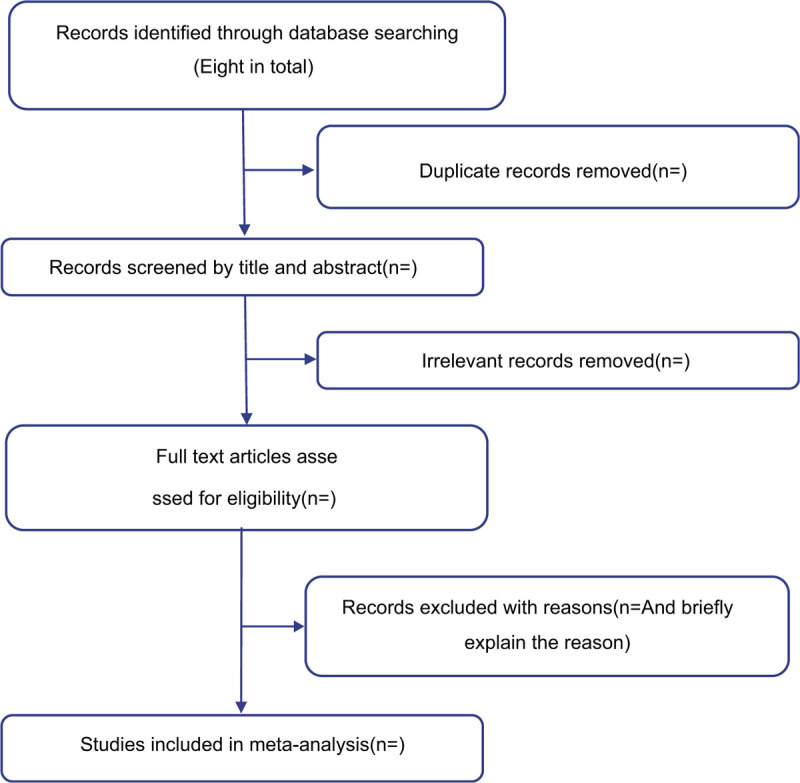
Flow diagram of study selection.

The retrieval will be carried out independently by 2 persons (SY and HD) according to the “retrieval method”, and the inspection results will be checked by the third team member (XW). After the check, the 2 personnel will respectively include the documents that meet the inclusion criteria through reading the title and abstract. In case of any difference, the 2 personnel will discuss and decide. If the discussion fails to resolve the difference, the third person‘s comments will be adopted. The reviewers will record the reasons for exclusion and show them in the Preferred Reporting Items for Systematic Review and Meta-Analysis Protocols flowchart (Fig. 1).

### 3.4 Data management assessment and methodological quality

An online reference management database, EndNote X9 will be used to manage the data. Selection bias, detection bias, performance bias, incomplete bias and other bias will be evaluated of each included study by 2 review authors independently. The Cochrane Risk of Bias Assessment Tool will be used to mark each bias as “YES” (low risk of bias), “NO (high risk of bias)”, or “UNCLEAR”(uncertain risk of bias).^[17]^ Any disagreements will be discussed with a third review author. If there is unclear information, it will be obtained by contacting the author.

### 3.5 Data synthesis and statistical analysis

Basic information about literature, baseline information and experimental results will be extracted, including article title; author details; year of journal publication; study location; study size; follow-up duration; age at baseline; sex distribution; duration of diabetes; endpoint definition and ascertainment and study outcomes of Montreal Cognitive Assessment scores.^[18]^ or Mini-mental State Examination score.^[19]^ Odds ratio and 95% confidence interval (CI) will be calculated to assess the association between DPP-4 inhibitors and cognitive impairment. If more than 3 RCTs are included, the state software will be used for meta-analysis. According to Cochrane Handbook,^[20]^*I*^2^ is less than 20% with fixed effect model, 20% to 50% with random effect model, and more than 50% with subgroup analysis. When there are more than 9 studies, a sensitivity analysis will be conducted.

### 3.6 Subgroup analysis

If sufficient data is available, the following subgroup analysis will be performed: the effect of different doses of treatment; patient demographics (age, sex, and severity of the disease); combined other diabetes complications.

### 3.7 Ethics and dissemination

The results of this meta-analysis and meta-regression will be disseminated through publication in a peer-reviewed journal and be presented at a relevant conference. The data that will be used will not contain individual patient data; therefore, there are no concerns about patients’ privacy.

## 4 Discussion

The present manuscript want to provide the protocol that will be used in the systematic review and meta-analysis to evaluate the role of DPP-4 inhibitors in cognitive impairment of T2DM patients and analyze the mechanism as much as possible. The existing studies have shown the potential effect of dpp-4 inhibitors, but there are still different opinions. The significance of a review and meta-analysis is to evaluate its effect in a larger sample, and to summarize the current research results for further to provide advice on clinical research, which will have a positive significance of protecting the cognitive impairment of diabetic patients.

## Author contributions

SYL conceived the idea and HD, YGX, XW designed the study. HD and XMY reviewed scoping searches and contributed to the methodological development of the protocol. SYL, YGX drafted the initial manuscript and all the authors (SYL, XW, HD, YGX, XMY, QC) revised the manuscript. All the authors have given approval for publishing. QC is the review guarantor.

**Data curation:** Huan DENG, Xiaomei YANG.

**Methodology:** Xian WANG, Huan DENG, Yuguo XIA, Xiaomei YANG.

**Writing – original draft:** Xian WANG, Yuguo XIA, Qiu CHEN.

**Writing – review & editing:** Qiu CHEN.

## Supplementary Material

Supplemental Digital Content
