# Supplementary material for: Effect of dipeptidyl peptidase-4 inhibitors inhibitor on cognitive dysfunction in diabetes: A protocol for systematic review and meta analysis
Source: Medicine (Baltimore). 2020 Jul 31;99(31):e20707. doi: 10.1097/MD.0000000000020707 (PMC7402785; doi:10.1097/MD.0000000000020707)
Supplement: Supplemental Digital Content [file medi-99-e20707-s001.docx]

Table 1

| **Search strategy to be used in Cochrane Library** |
| --- |
| **number search terms** |
| 1 Cognitive Dysfunction [MeSH] |
| 2 Dementia [MeSH] |
| 3 Cognitive Dysfunctions OR Dysfunction, Cognitive OR Dysfunctions, Cognitive OR Cognitive Impairments  OR ... (Search all free words) |
| 4 Dementias OR Amentia OR Amentias OR  SenileParanoid Dementia OR ... (Search all free words) |
| 5 1 OR 2 OR 3 OR 4 |
| 6 Dipeptidyl-Peptidase IV Inhibitors [MeSH] |
| 7 Dipeptidyl Peptidase IV Inhibitors OR Inhibitors, Dipeptidyl-Peptidase IV OR Gliptins OR ...:ti,ab (Search all free words) |
| 8 6 OR 7 |
| 9 Mental Status and Dementia Tests [MeSH] |
| 10 General Practitioner Assessment of Cognition OR GPCOG OR Montreal Cognitive Assessment  OR ...ti,ab (Search all free words) |
| 11 9 OR 10 |
| 12 5 AND 8 |
| 13 8 AND 9 |
